# Injury Profile SIMulator, a Qualitative Aggregative Modelling Framework to Predict Injury Profile as a Function of Cropping Practices, and Abiotic and Biotic Environment. II. Proof of Concept: Design of IPSIM-Wheat-Eyespot

**DOI:** 10.1371/journal.pone.0075829

**Published:** 2013-10-16

**Authors:** Marie-Hélène Robin, Nathalie Colbach, Philippe Lucas, Françoise Montfort, Célia Cholez, Philippe Debaeke, Jean-Noël Aubertot

**Affiliations:** 1 Institut National de la Recherche Agronomique, Unité Mixte de Recherche 1248 Agrosystèmes et agricultures, Gestion des ressources, Innovations et Ruralités, Castanet-Tolosan, France; 2 Université de Toulouse, Institut National Polytechnique de Toulouse, Ecole d'Ingénieurs de Purpan, Toulouse, France; 3 Institut National de la Recherche Agronomique, Unité Mixte de Recherche 1347 Agroécologie, Dijon, France; 4 Institut National de la Recherche Agronomique, Unité Mixte de Recherche 1099 Biologie des Organismes et des Populations appliquée à la Protection des Plantes. Le Rheu, France; 5 Université Toulouse, Institut National Polytechnique de Toulouse, Unité Mixte de Recherche 1248 Agrosystèmes et agricultures, Gestion des Ressources, Innovations et Ruralités, Castanet-Tolosan, France; University of Florida, United States of America

## Abstract

IPSIM (Injury Profile SIMulator) is a generic modelling framework presented in a companion paper. It aims at predicting a crop injury profile as a function of cropping practices and abiotic and biotic environment. IPSIM's modelling approach consists of designing a model with an aggregative hierarchical tree of attributes. In order to provide a proof of concept, a model, named IPSIM-Wheat-Eyespot, has been developed with the software DEXi according to the conceptual framework of IPSIM to represent final incidence of eyespot on wheat. This paper briefly presents the pathosystem, the method used to develop IPSIM-Wheat-Eyespot using IPSIM's modelling framework, simulation examples, an evaluation of the predictive quality of the model with a large dataset (526 observed site-years) and a discussion on the benefits and limitations of the approach. IPSIM-Wheat-Eyespot proved to successfully represent the annual variability of the disease, as well as the effects of cropping practices (Efficiency = 0.51, Root Mean Square Error of Prediction = 24%; bias = 5.0%). IPSIM-Wheat-Eyespot does not aim to precisely predict the incidence of eyespot on wheat. It rather aims to rank cropping systems with regard to the risk of eyespot on wheat in a given production situation through *ex ante* evaluations. IPSIM-Wheat-Eyespot can also help perform diagnoses of commercial fields. Its structure is simple and permits to combine available knowledge in the scientific literature (data, models) and expertise. IPSIM-Wheat-Eyespot is now available to help design cropping systems with a low risk of eyespot on wheat in a wide range of production situations, and can help perform diagnoses of commercial fields. In addition, it provides a proof of concept with regard to the modelling approach of IPSIM. IPSIM-Wheat-Eyespot will be a sub-model of IPSIM-Wheat, a model that will predict injury profile on wheat as a function of cropping practices and the production situation.

## Introduction

Stem base diseases on cereals and grasses are widespread in many eco-regions of the world and cause important production and economic losses. The most detrimental foot and root pathogens on cereals in temperate areas are *Pseudocercosporella herpotrichoides*; *Fusarium* spp, *Rhizoctonia cerealis* and *Gaeumannomyces graminis* [1]. Eyespot caused by the necrotrophic and soil-borne fungi *Oculimacula yallundae* and *O. acuformis*, anamorph *Pseudocercosporella herpotrichoides* [2]–[4] is considered to be the most important stem base disease of cereals in temperate countries [5]. Under cool and wet conditions in autumn and spring, both species sporulate and infect the stem bases of their hosts. Without any host crops (cereals, ryegrass), the pathogen survives on previously infected stubble, on which splash-dispersed conidia and air-dispersed ascospores are produced [6]. Injuries interfere with the circulation of nutrients and water through the base of the stem [7] leading to a weakening and possibly to a breakage of the stem base, causing lodging before harvest [5], [8]. Relative yield losses of up to 50% have been reported for the most severe attacks on winter wheat with lodging [2], [7], [9]–[11].

In the past, the control of eyespot has relied largely on chemical protection [12]. However, due to the development of resistance to the main available fungicides in *O. yallundae* and *O. acuformis* populations, adaptation of the entire cropping system to control eyespot on wheat is a sound alternative [13], [14]. Furthermore, growing concerns about the impact of pesticides on the environment and human health has led to attempts to limit pesticide use [15], [16]. Most governments of developed countries have launched national action plans to reduce pesticide use. For instance, the French government has set as a goal to reduce pesticide use by 50% by 2018 if possible [17]. The European Union has proposed to encourage the use of low-pesticide farming as one of its priorities by the Sustainable Use Directive (SUD) (http://eurlex.europa.eu/LexUriServ/LexUriServ.do?uri=OJ:L:2009:309:0071:0086:FR:PDF, accessed November 2012).

In addition, the USA decided to support and develop Integrated Pest Management (IPM) nationwide in order to reduce pesticide use [18]. It appears necessary therefore to combine various methods (cultural, genetic and chemical) in IPM strategies [19] to control eyespot on wheat. The main cultural practices that can partly control eyespot through a specific adaptation are: a low host frequency in the crop sequence, infected stubble management through adapted tillage, a late sowing date and low sowing rate [10], [20], [29] The genetic control of eyespot consists of using resistant cultivars. There are several known sources of resistance to eyespot, but only three resistance genes have been described so far [21]–[23].

IPM strategies, based on these control methods, have to be developed, adapted and applied to a wide range of physical, chemical, biological and socio-economic contexts. However, it is extremely difficult to describe the entirety of the cropping practices*environment*crop*pest system because of the tremendous number of interactions [24]. Modelling is certainly the best way to handle such a level of complexity and to help design sustainable innovative cropping systems less reliant on pesticides.

However, crop models do not deal with injuries caused by pests [25] and few pest models integrate the effects of cultural practices because of the difficulty of describing their numerous consequences on the agroecosystem [26] Thus, different models have been developed to represent eyespot injuries on wheat [27]–[29] or the associated damage [30] Among these, only one model takes into account the effect of the cropping system (crop succession, tillage, sowing date, sowing rate, total nitrogen fertiliser and its form) on injuries caused by eyespot [29]. However, this model does not take into account soil and climate, along with some cultural practices that can greatly influence the disease development (e.g. cultivar choice). There is therefore a need for a model that predicts as exhaustively as possible the effect of cropping practices on eyespot on wheat in a given production situation.

In this article, we will define the production situation as the physical, chemical and biological components, except for the crop, of a given field (or agroecosystem), its environment, as well as socio-economic drivers that affect farmers' decisions (adapted from [31], [32], [33]). In this definition, “environment” refers to climate and the fraction of the territory that can influence pest dynamics through dispersal of harmful or beneficial organisms. In a given production situation, a farmer can design several cropping systems according to his goals, his perception of the socio-economic context and his environment, farm features, his knowledge and cognition. However, it is assumed that a given cropping system in a given production situation, such as defined above, should lead to a unique injury profile. In IPSIM, production situations are partly described by three components: soil, climate, and the biological environment of the field [33]. In the approach used here, the farmer's decision-making process and socio-economic drivers are not taken into account.

The conceptual bases of IPSIM have been described in detail by Aubertot and Robin [33]. The generic hierarchical aggregative modelling framework of IPSIM aims at predicting an injury profile as a function of cropping practices, soil, and climate and the biological field environment for any mono-specific crop production (arable crop, perennial or protected crops). In order to test whether this modelling approach could be successfully applied to represent injuries caused by a single pest, a model, named IPSIM-Wheat-Eyespot, has been developed according to the conceptual framework of IPSIM. It aims at predicting the final incidence of eyespot on wheat as a function of the production situation and cropping practices. IPSIM-Wheat-Eyespot gathers available knowledge in the scientific literature (models, experimental results) and expertise and will help design cropping systems with low risk of eyespot on wheat and perform diagnoses of commercial wheat fields. IPSIM-Wheat-Eyespot will be used as a sub-model for IPSIM-Wheat, a model that will predict the injury profile on winter wheat (i.e. the distribution of injuries caused by the most important detrimental pests on wheat [34]). This paper presents the method used to develop IPSIM-Wheat-Eyespot using the conceptual modelling framework of IPSIM [33], an evaluation of its predictive quality and a discussion on the limitations and benefits of the model.

## Materials and Methods

### Design of IPSIM-Wheat-Eyespot

#### 1. General Approach

IPSIM-Wheat-Eyespot is based on the DEX method, and is implemented with the software DEXi [35]. DEX is a method for qualitative hierarchical multi-attribute decision modelling and support, based on a breakdown of a complex decision problem into smaller and less complex sub-problems, characterised by indicators (or attributes) that are organised hierarchically into a decision tree. These attributes are characterised by their name, a description and a scale. DEXi is generally used to evaluate and analyse decision problems, e.g. [36]. However, the DEX method has been used here in an original way to model complex agroecosystems. IPSIM-Wheat-Eyespot is therefore a hierarchical and qualitative multi-criteria model, allowing the prediction of eyespot injury according to various factors with sometimes opposite effects. IPSIM-Wheat-Eyespot has the following features (derived from [37]):

- Processes are hierarchically organised into a tree of attributes that constitutes the structure of the model;
- Terminal attributes of the tree (i.e. leaves or *basic attributes*) are input variables of the model and must be specified by users; the “trunk” of the tree (i.e. the final aggregated attribute) is the main model output variable (final eyespot incidence on wheat); internal nodes are called *aggregated attributes*;
- All model attributes are qualitative variables (nominal or ordinal) rather than quantitative variables. They take only discrete symbolic values, usually represented by words rather than numbers: e.g. “ploughing, stubble disking, rotary harrowing” for nominal variables, “low, medium, high” for ordinal variables;
- The aggregation of values up the tree is defined by aggregating tables for each aggregated attribute based on “if-then” decision rules. These aggregating tables can be seen as equivalents of parameters for quantitative numerical models, whereas the tree of attributes can be viewed as the equivalent of their mathematical structure.

IPSIM-Wheat-Eyespot was designed in 3 steps [37]: (1) identification and organisation of the attributes, (2) definition of attribute scales, and (3) definition of aggregating tables.

#### 2. Identification and Organisation of Attributes

IPSIM-Wheat-Eyespot aims at predicting the incidence of eyespot on wheat in a given field according to a set of input variables. The spatial scale addressed is the field and the temporal scale is the wheat growing season, although some input variables encompass the crop sequence (up to the pre-preceding crop). IPSIM-Wheat-Eyespot is a static deterministic model.

**Figure 1:**
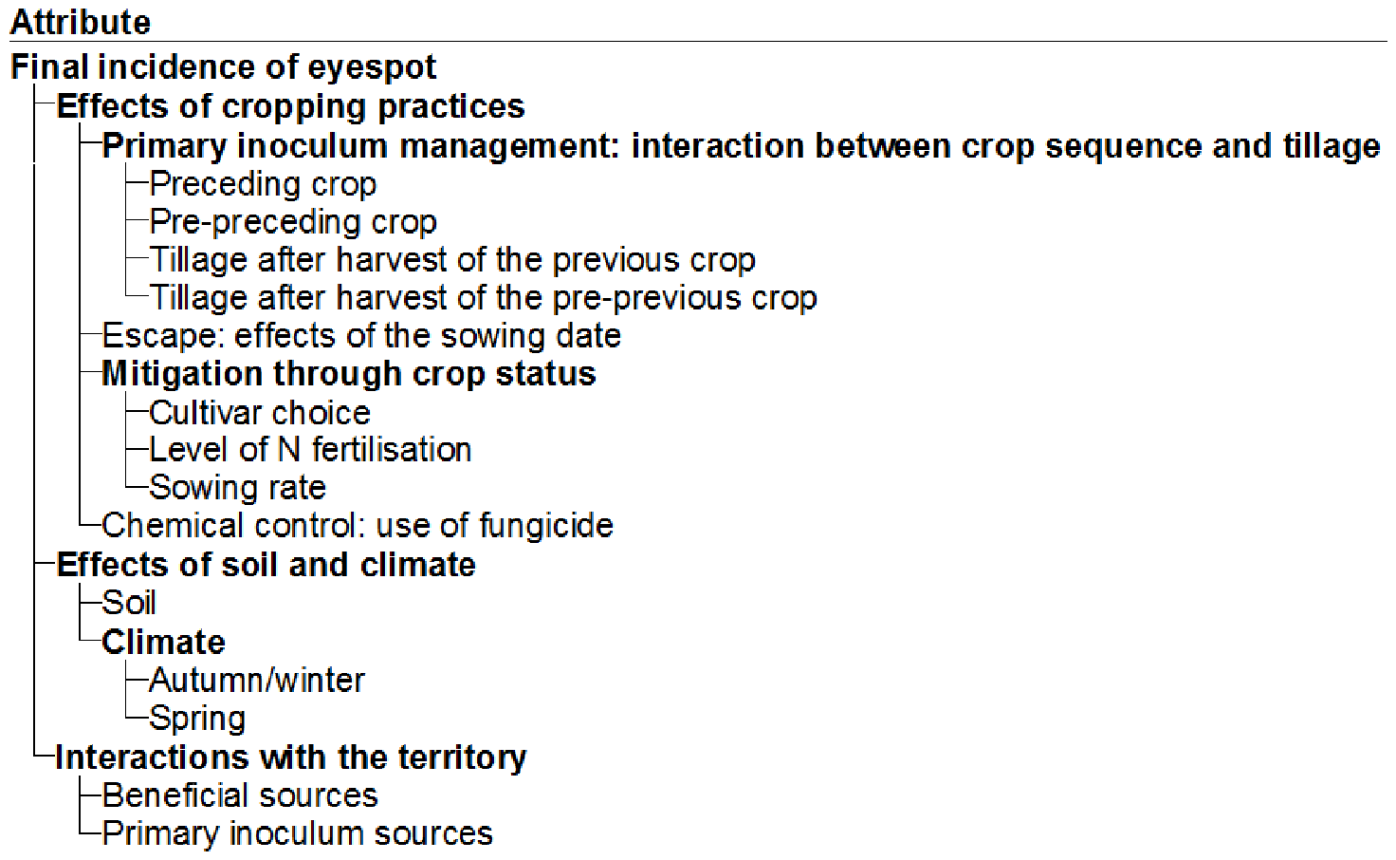
Hierarchical structure of IPSIM-Wheat-Eyespot (screenshot of the DEXi software). Bolded and non-bold terms represent aggregated and basic attributes, respectively.

**Figure 2:**
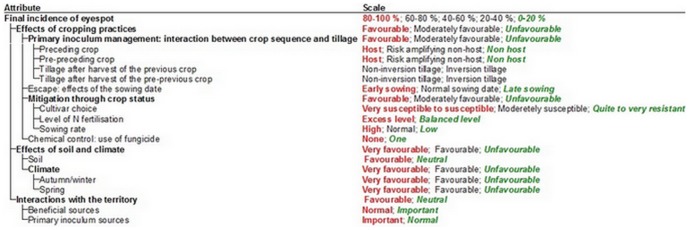
Attribute scales of IPSIM-Wheat-Eyespot (screenshot of the DEXi software). All the scales are ordered from values detrimental to the crop (i.e. favourable to eyespot) on the left-hand side to values beneficial to the crop on the right-hand side (i.e. unfavourable to eyespot). In the DEXi software, this difference is clearly visible because, by convention, values beneficial to the user are coloured in green, detrimental in red, and neutral in black.

The hierarchical structure presented in Figure 1 represents the breakdown of factors affecting eyespot final incidence into specific explanatory variables, represented by lower-level attributes. This figure represents the adaptation to eyespot of the model structure presented in Figure 2 by Aubertot and Robin [33].

In all, IPSIM-Wheat-Eyespot has 21 attributes, of which 14 are basic (i.e. input variables) and 7 aggregated. The 14 basic attributes are presented as the terminal leaves of the tree and their levels are aggregated into higher levels according to aggregating tables. They represent input variables of the model. Some of them (e.g. those representing the interactions at the territory level) could be omitted since they do not influence the final output. However, they were kept because these basic attributes will be necessary for the modelling of the whole injury profile on wheat. The aggregated attributes are internal nodes. They represent state variables or the output variable of IPSIM-Wheat-Eyespot. They are determined by lower-level basic attributes [38]. The output of IPSIM-Wheat-Eyespot is represented by the attribute “Final eyespot incidence” (eyespot incidence at the “milky grain”, stage 7: development of fruit on BBCH scale [39]) which is determined by three main factors: cropping practices, soil and climate and the biological environment of the considered field. This is reflected by the hierarchical structure of the model, which consists of three sub-trees of attributes (Figure 1) split into one main part and two smaller ones. The main sub-tree, “Effect of cropping practices”, illustrates the complexity of the effects of cropping practices and the need to consider a combination of practices in order to evaluate the final eyespot incidence. It uses indicators based on tactical (with a short time-frame) or strategic decisions (with a longer time-frame [40]). These decisions can affect the agroecosystem at several stages.

- Eyespot is considered as a highly endocyclic disease (as defined in [33]). Upstream, some cropping practices affect the quantity of the endo-inoculum (initial pathogen population present in the field). Crop sequence and tillage determine the vertical distribution of infected stubble and have proven to be of major importance for eyespot control [41]–[45]. Nevertheless, the effects of tillage on the disease are controversial in the literature. According to several authors [1], [41], [46]–[50], minimum tillage is highly favourable to eyespot development in the presence of preceding host-crop residues in the top layer, whereas ploughing significantly reduces its incidence by burying host-crop residues. These results conflict with those that show that eyespot was more severe after soil inversion than after non-inversion under moist, cool conditions [44]–[47], [51]–[55]. The possible explanation of this apparent contradiction is that non-inversion is more favourable to antagonistic micro-organisms than ploughing (the microbiological activity is higher at the soil surface than in the top 20 cm soil layer and the weather in some experiments, such as those in Italy, was probably too dry for antagonistic biota to flourish on crop debris and thus to control eyespot [1].
- Action by escape consists of shifting periods of highest crop susceptibility away from the main periods of pathogen contamination. This is achieved by altering the wheat sowing date. In the case of eyespot, “escape strategies” cannot really be considered. However, early sowing increases the probability of autumn contamination through primary infection, due to the longer time available for eyespot to develop and to affect stems [42].
- During the crop cycle, some cropping practices can mitigate infection through crop status by increasing crop competitiveness and/or by creating less favourable conditions for pest development. Low plant density can limit pathogen development through several mechanisms, such as restricting the contact between plant organs and infectious propagules and lowering the humidity within the canopy. This results in a control of soil-borne diseases like eyespot by low plant density and/or a high shoot number per plant [20]. In addition, low densities increase distances between plants, which limits secondary pathogen cycles, and leads to a drier microclimate. Excessive use of nitrogen fertilisers produces lush crops and favours eyespot through direct and indirect effects [56], [57]. However, in the case of eyespot, nitrogen availability in the soil seems to be a minor factor for the development of the disease [10], [20], [29].
  Use of disease-resistant cultivars provides an economic, environmentally friendly and effective strategy to control disease. However, not all resistant cultivars have been assessed in integrated cropping systems [58] and cultivars do not share the same susceptibilities to different diseases [59]. Eyespot resistance is generally not complete and its expression depends widely on environmental factors [22].
- Lastly, a fall-back solution (use of fungicide) can be used when alternative practices are not sufficient. However, several studies have provided evidence for reduced susceptibility to fungicides in populations of *O. yallundae* and *O. acuformis* [60]. For the sake of simplicity, resistance to fungicide in pathogen populations was not taken into account in IPSIM-Wheat-Eyespot.

The two other sub-trees describe the biological environment of the considered field, as well as soil and climate. These sub-trees are not affected by cropping practices. Among these factors, climate is the main factor affecting eyespot development [9], [43].

#### 3. Definition of the Attribute Scales

The second step in the design of a DEXi model is the choice of ordinal or nominal scales for basic and aggregated indicators. Sets of discrete values were defined for all attributes of the model and described by symbolic value scales defined by words. These values were defined according to the knowledge available in the international literature and some expertise when needed. IPSIM-Wheat-Eyespot uses at most a three-grade value scale (i.e. “Unfavourable”, “Favourable”, “Very favourable”) for the aggregated and basic attributes. This scale refers to the disease. The value “Favourable” means that the attribute is favourable to the development of the disease and therefore potentially detrimental to the crop.

Some values for basic indicators can be specified using quantitative values that are then translated into qualitative values. For instance, the translation into qualitative values of the sowing date, sowing density or N rate is performed using experimental references or expertise. This translation takes into account the regional context. For example, a sowing date classified as “Early” in the south of France might be classified as “Normal” in northern France. This classification actually depends on the sowing date distribution in the considered region.

Other attributes are directly qualitatively estimated. For instance, the indicators “inversion tillage or non-inversion tillage” or “preceding and pre-preceding crop” are nominal variables and directly monitored as such in experiments [61], [62]. The level of cultivar resistance has been described using the official list provided by the French National Seed Station (Groupe d'Etude et de contrôle des Variétés et des Semences; http://cat.geves.info/Page/ListeNationale; accessed November 2012) and published by Arvalis-Institut du végétal (http://www.arvalisinfos.fr/_plugins/WMS_BO_Gallery/page/getElementStream.html?id=13504&prop=file; accessed November 2012). In this list, cultivars are rated for their susceptibility to eyespot on a 0–9 scale, from very susceptible to resistant.

For the climate attribute, a three-value scale (“Unfavourable”; “Favourable”; “Very favourable”) was defined using climatic models [27], [43] and data from the INRA Climatik database.

All the scales in Figure 2 are ordered from values detrimental to the crop (i.e. favourable to the disease) on the left-hand side to values beneficial to the crop on the right-hand side (i.e. unfavourable to the disease). In the DEXi software, this difference is clearly visible because, by convention, values beneficial to the user are coloured in green, detrimental in red, and neutral in black. The scales for the “tillage after preceding crop” and “tillage after pre-preceding crop” attributes appear in black since their effects on the disease cannot be defined independently from the crop sequence.

Initial input attribute values (either quantitative or qualitative) are translated into qualitative appreciation, according to two to three scales defined on the basis of available information in the literature, models or expertise. Sometimes, a two-value scale is enough to represent the value of an indicator (e.g. chemical control was applied or not; or the soil has either been ploughed or not after the preceding harvest). However, other attributes usually need a three-value scale to describe the diversity of cropping practices or environment (e.g. the sowing rate attribute requires three grades to describe farmers' practices: the sowing rate can be low, normal or high).

#### 4. Definition of Aggregating Tables

**Figure 3:**
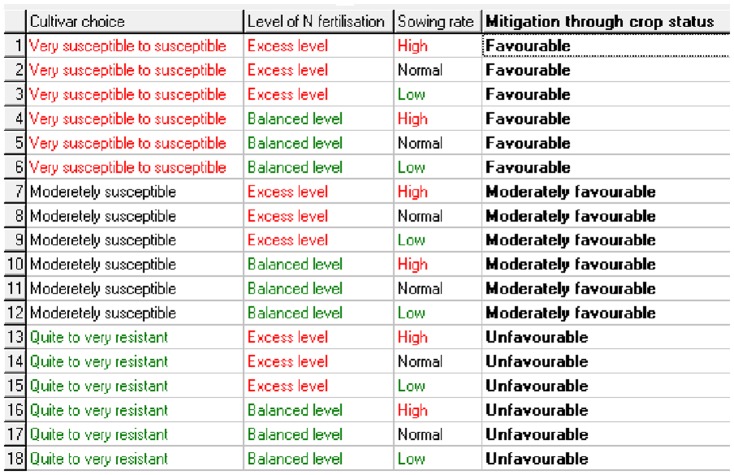
Aggregating table for the “Mitigation through crop status” aggregated attribute (screenshot of the DEXi software). Aggregation rules for the 18 possible combinations of the 3 cultivar choices, the 2 levels of fertilisation and the 3 sowing rates.

The third step in the design of a DEXi model is the choice of aggregating tables determining the aggregation of attributes in the tree and their interactions. For each aggregated attribute in the model, a set of “*if-then*” rules define the value of the considered attribute as a function of the values of its immediate descendants in the model. The rules that correspond to a single aggregated attribute are gathered together and conveniently represented in tabular form. In this way, each table defines a mapping of all value combinations of lower-level attributes into the values of the aggregate attribute. Figure 3 shows decision rules that correspond to the “mitigation through crop status” aggregated attribute and define the value of this attribute for the 18 possible combinations of the three cultivar choices, the 2 levels of fertilisation and the 3 sowing densities. For example, if the cultivar is quite resistant, the level of N fertilisation balanced and the sowing rate low, then the “mitigation through crop status” attribute will be unfavourable to eyespot (the final incidence will decrease). However, even if the sowing rate and the N application rate are both high, the “mitigation through crop status” attribute during wheat growth will control eyespot significantly because the “cultivar choice” attribute is much more influential than the two other attributes (Figure 3).

The aggregating tables of IPSIM-Wheat-Eyespot have been established using knowledge available in the international literature and summarised in Table 1, and expert knowledge when needed. All aggregating tables of the model are presented in figures S1, S2, S3, S4, S5, S6.

**Table 1:** Available knowledge in the scientific literature describing the effects of cropping practices and the production situation on the incidence of eyespot on wheat.

Factor	Direction of the effect	Intensity of the effect	Impact on eyespot development	References
Tillage	+/−	++	Contradictory results. For some authors, reduced soil tillage decreased eyespot infection. For others, eyespot was often more severe after ploughing than after non-inversion tillage.	[1], [11], [41], [42], [44]–[55]
Preceding and pre-preceding crop	+	++	Preceding and pre-preceding host crops are known to favour eyespot. However, the interaction between tillage and the crop sequence has to be taken into account.	[9], [10], [29], [41]–[42], [55], [61], [62]
Sowing date	+	++	Eyespot has always been reported to be more severe in early sown crops.	[10], [20], [27], [29], [42], [55]
N fertilisation rate	+	+	High nitrogen availability generally favoured the disease. However these results were questioned.	[9], [10], [20], [29], [56], [57]
Sowing rate	+	+	Prevalence was increased by high plant density and/or low shoot number per plant.	[20], [29]
Cultivar choice	+	+++	The use of varieties with resistance could obviate the need for fungicide.	[10], [21], [22], [42], [58], [59]
Cultivar mixture	0	0	No significant difference was found between the disease level in mixtures and the mean of disease level of the mixture components in pure stands.	[70]–[72]
Climate	+	++	Eyespot strongly depends on climate. Infections require periods of at least 15 h with T° between 4°C and 13°C and HR>80% (from October to April).	[9], [27], [28], [29], [43]

Cropping practices and climate can be favourable (+), unfavourable (−) or neutral (0) to the development of eyespot. The intensity of the considered factor is summarised with 4 classes: 0, no effect; +, slight; ++, significant; +++, crucial.

#### 5. Attribute Weights

The influence of each basic and aggregated attribute on the value of the output variable can be characterised with weights. The higher the weight, the more important the attribute. Table 2 summarises the weights of each of the 19 attributes of the model, providing an overview of the model's structure. IPSIM-Wheat Eyespot has 3 levels of aggregation (Figure 1), the third one being the leaves (i.e. the model input basic attributes). The “local” and “global” weights are normalised in two different ways. “Local” weights are given to each aggregated attribute separately so that the sum of weights of its immediate descendants in the hierarchy equals 100%. The “global” weights are calculated at a given level of aggregation and express the influence of each attribute at that aggregation level. They are obtained by multiplying the local weight of a given attribute at a given level of aggregation, by local weighting of its ascendants. For instance, the value of the “soil and climate” attribute is completely defined by the “Climate” attribute (100%, local weight), but this attribute only contributes 53% to the definition of the value of “Eyespot incidence” (global weight at the second level of aggregation). Local and global weights are identical at the first level of aggregation, since in this case there is only one level of aggregation. Global weights of basic attributes are shown in bold in Table 2 in order to ease their identification, since they are distributed among the second and third levels of aggregation of IPSIM-Wheat-Eyespot. The sum of global weights at the third level is only 76%. This is because some basic attributes are directly embedded in the model at the second level of aggregation. The sum of global basic attribute weights is logically equal to 100%. Table 2 can be seen as an equivalent of a sensitivity analysis that would aim at identifying the most influential input (and state) variables of a quantitative model.

**Table 2:** Respective weights of the attributes of IPSIM-Wheat-eyespot.

Attributes defining the final incidence of eyespot	Local level 1	Local level 2	Local level 3	Global level 1	Global level 2	Global level 3
1 Effects of cropping practices	47			47		
1.1 Primary inoculum management		21			10	
**1.1.1 Preceding crop**			40			4
**1.1.2 Pre-preceding crop**			12			1
**1.1.3 Tillage after the preceding crop**			40			4
**1.1.4 Tillage after pre-preceding crop**			8			1
**1.2 Escape: effects of sowing date**		9			4	
1.3 Mitigation through crop status		26			12	
**1.3.1 Cultivar choice**			100			12
**1.3.2 Level of N fertilisation**			0			0
**1.3.3 Sowing rate**			0			0
**1.4 Chemical control**		44			21	
2 Effects of soil and climate	53			53		
2.1 Soil		0			0	
2.2 Climate		100			53	
**2.2.1 Autumn/winter**			29			15
**2.2.2 Spring**			71			38
3 Interactions with the rest of the territory	0			0		

The “local” and “global” weights are calculated for each aggregated attribute separately and are distributed in 3 levels of aggregation. Bold and non-bold terms represent basic attributes and aggregated terms, respectively.

#### 6. Simulations with DEXi

**Figure 4:**
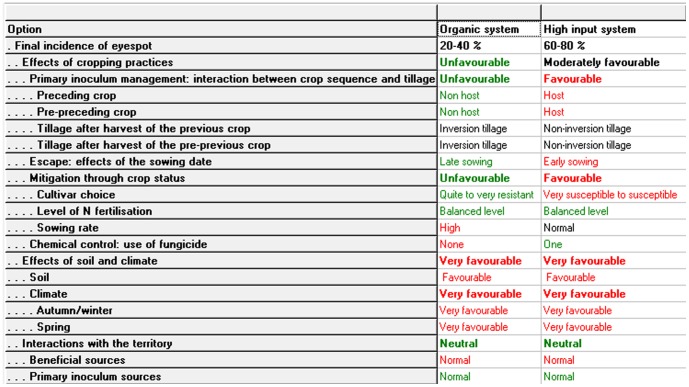
Example of 2 simulations carried out with IPSIM-Wheat-Eyespot (screenshot of the DEXi software).

The qualitative final attribute value (final incidence of eyespot) is calculated by DEXi. The calculation consists in computing all aggregated attribute values according to: (i) the structure of the tree; (ii) a set of input variables (basic attribute values) defining a simulation unit; and (iii) the aggregating tables for the aggregation of attributes. An example of output results obtained for two simulation units is provided in Figure 4 (input basic attributes and calculated aggregated attribute values for the simulation of two systems: an organic and a high-input one).

### Evaluation of the Predictive Quality of IPSIM-Wheat-Eyespot

#### 1. Description of the Dataset Used

Data representative of a wide range of climate patterns, soils and cropping practices are needed to assess the predictive quality of the model. A large dataset was therefore developed to assess the predictive quality of IPSIM-Wheat-Eyespot. A national survey was conducted to identify relevant data from various research and development institutes. The required datasets had to provide information for input attributes of IPSIM-Wheat-Eyespot (description of cropping practices, soil and climate) and its output (eyespot incidence at the “milky grain”, stage 7: development of fruit on BBCH scale [39]). The dataset obtained is summarised in Table 3. It comprises results from multifactorial trials from 1980 to 1994 in 7 contrasting regions in France, which were set up to analyse the effects of various cropping practices on foot and root winter wheat diseases on different soils and with differing climate patterns. Various cultivars were combined with different crop sequences, conventional and reduced tillage, low or high plant densities, early or late sowing dates, low or high N fertilisation, in various areas of production where eyespot epidemics are observed. Most of these trials were specific studies on foot diseases [20], [41], [61], [62], so the experimental conditions were suited to ensure the presence of eyespot (i.e. infected wheat present in the crop sequence and only susceptible cultivars). Other data originated from a regional agronomic diagnosis [63] performed in cereal fields from 1987 to 1994 in 19 French regions to analyse the effects of cultural practices on the incidence and severity of foot and root disease complexes [64]. In this survey, data were collected on 894 cereal fields in a wide range of production situations.

**Table 3:** Main features of the datasets used for the evaluation of IPSIM-Wheat-Eyespot's predictive quality.

Cropping practice	Design	Year	Location	Number of site-years	References
Crop sequence	Multifactorial field trials	1981–1982	Toulouse (Midi-Pyrénées)	11	[61]
Crop sequence including various durations of continuous cereal cropping	Multifactorial field trials	1980–1994	Grignon (Ile-de-France)	29	[62]
Tillage (soil structure)	Multifactorial field trials	1992–1993	Péronne (Picardie)	8	[73]
Tillage (crop residue vertical distribution)	Multifactorial field trials	1992–1993	Chartres (Centre), Grignon (Ile-de-France)	12	[41]
Sowing date, sowing rate, N fertilisation	Multifactorial field trials	1992–1994	Chartres, La Verrière (Ile-de-France), Le Rheu (Bretagne), Nancy (Lorraine), Dijon (Bourgogne)	95	[20]
Tillage, previous crop, fertilisation, sowing rate, sowing date, cultivar choice and use of fungicide	Diagnoses in cereal fields	1987–1994	19 French regions	370	[64]
Crop sequence	Multifactorial field trials	1981–1982	Toulouse (Midi-Pyrénées)	11	[61]
Crop sequence including various durations of continuous cereal cropping	Multifactorial field trials	1980–1994	Grignon (Ile-de-France)	29	[62]
Tillage (soil structure)	Multifactorial field trials	1992–1993	Péronne (Picardie)	8	[73]
Tillage (crop residue vertical distribution)	Multifactorial field trials	1992–1993	Chartres (Centre), Grignon (Ile-de-France)	12	[41]
Sowing date, sowing rate, N fertilisation	Multifactorial field trials	1992–1994	Chartres, La Verrière (Ile-de-France), Le Rheu (Bretagne), Nancy (Lorraine), Dijon (Bourgogne)	95	[20]
Tillage, previous crop, fertilisation, sowing rate, sowing date, cultivar choice and use of fungicide	Diagnoses in cereal fields	1987–1994	19 French regions	370	[64]

For some situations, the pre-preceding crop (3 possible types of crop in the model: “host”, “non-host” and “risk amplifying”) and the associated tillage after the harvest of this crop (2 possible values in the model) were not observed. Instead of ignoring these precious data, simulations were performed for the 3*2 possibilities and only cases for which the 6 simulations led to similar output values were kept for evaluating the model. In all, 526 site-years were used for the evaluation of the model and they represented a large number of combinations of cropping practices and production situations (19 French regions over 9 years).

The data presented in Table 3 were transformed into qualitative values and used as input basic attributes to feed IPSIM-Wheat-Eyespot.

#### 2. Evaluation of the Predictive Quality of IPSIM-Wheat-Eyespot

The evaluation consisted in comparing simulated and observed values. Since the model predicts classes of incidence, observed incidences at wheat stage 7 were transformed into observed incidence classes using the same discretisation as the model (i.e. 0–20%, 20–40%, 40–60%, 60–80%, 80–100%). However, one might want to predict incidences rather than classes of incidence. In order to test the predictive quality of IPSIM-Wheat-Eyespot for incidences, its output main variable was transformed into a numerical value by replacing the predicted incidence class by the centre of the class. The model was therefore evaluated in two ways: first, on its ability to predict incidence classes, and second on its ability to predict eyespot incidences.

For incidence classes, the deviation of the model was characterised by calculating the number of classes of difference between observed and simulated classes. The distribution of simulated classes was displayed according to observed incidence classes. This information was summarised by a multinomial distribution in 9 difference classes (from −4 to +4) since the model has 5 incidence classes. The proportion of situations for which the model correctly predicted the observed incidence class was taken as an indicator of the quality of prediction of the model. In addition, a non-parametric Wilcoxon test was performed to test whether the distribution of errors was zero-centred (in that case, the model can be considered unbiased).

For incidences, the predictive quality of IPSIM-Wheat-Eyespot was characterised using three common statistical criteria [65]: bias (Equation 1), Root Mean Square Error of Prediction (RMSEP, Equation 2), and efficiency (Equation 3).
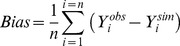
(1)where n is the total number of considered situations, *Y_i_^obs^* the observed value for situation *i*, and *Y_i_^sim^* is the corresponding value simulated by the model. The bias measures the average difference between observed and simulated values. If the model underestimates the considered variable, the bias is positive. Conversely, if the model overestimates the variable, the bias is negative.
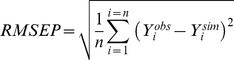
(2)RMSEP quantifies the prediction error when the model parameters have not been estimated using the observations *Y_i_^obs^* used in the calculation of this criterion.
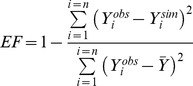
(3)Where 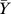 is the mean of observed data. Nash and Sutcliffe [66] defined the efficiency as a normalised statistic that determines the relative magnitude of the residual variance (“noise”) compared with the measured data variance (“information”). The efficiency defines the ability of a model to predict the value of a variable. The efficiency can range from –∞ to 1. If the model perfectly predicts the observations, the efficiency is maximum and is equal to 1. Efficiency values lower than 0 indicate that the mean observed value is a better predictor than the simulated values, which indicate a poor predictive quality of the model. Values between 0 and 1 are generally viewed as acceptable levels of performance. The closer the model efficiency is to 1, the better is the fit between observed and simulated data [65].

## Results

### Evaluation of the Quality of Prediction for Final Incidence Classes

**Figure 5:**
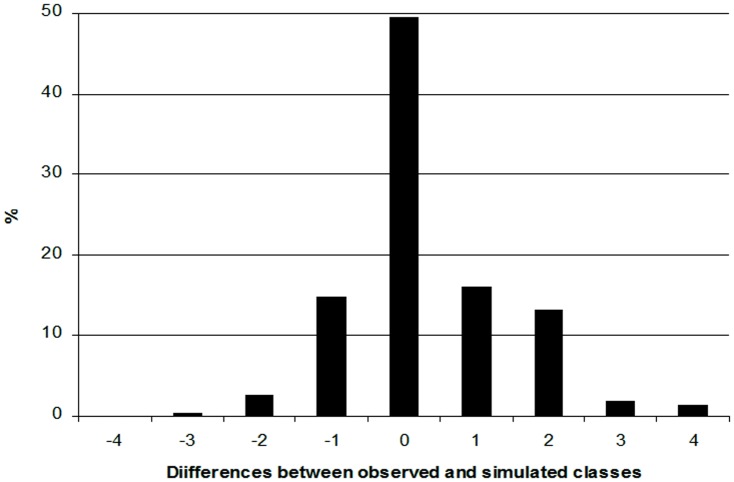
Evaluation of the predictive quality of IPSIM-Wheat-Eyespot. Residuals distribution: number of classes of difference between observed and simulated final eyespot classes (0–20%, 20–40%, 40–60%, 60–80%, 80–100%; 526 fields, over 9 years and 19 French regions).

**Figure 6:**
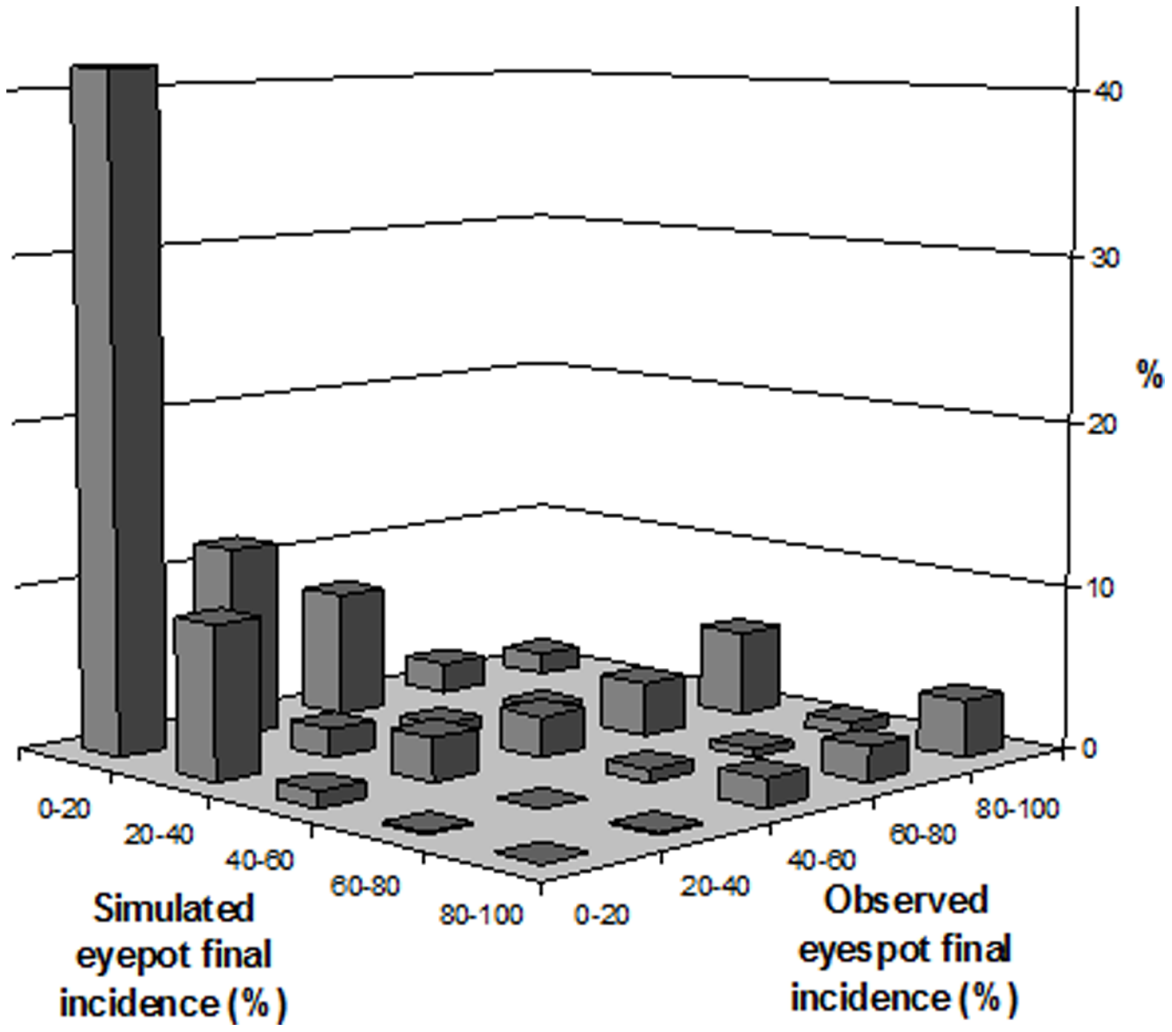
Evaluation of the predictive quality of IPSIM-Wheat-Eyespot Distribution of class differences between observed and predicted final eyespot incidences. (526 fields, over 9 years and 19 French regions).

The high number of observed site-years in the dataset (526) permitted a reliable evaluation of the predictive quality of IPSIM-Wheat-Eyespot. Residuals were distributed around 0 (Figure 5), indicating that the predicted values were close to observations. Nearly half (47.1%) of the simulated classes encompassed the observed values and 80.4% had at most a difference of one class only. In addition, there are nearly as many negative as positive differences of exactly one class. The Wilcoxon test performed over the 9 class differences (from −4 to +4) proved that the model was significantly biased (simulated final incidence classes lower than observations, p<1.0 10^−10^). Figure 6 illustrates the distribution of class differences between observed and predicted final eyespot incidences. The overall predictive quality of IPSIM-Wheat-Eyespot was judged fair, even if slightly biased. The predictive quality was good for the lowest class (52% of all the observations in the dataset): 80% of the observed values between 0 and 20% were correctly simulated. The model underestimated final incidences for observations higher than 20%.

### Evaluation of the Quality of Prediction for Final Incidence Values

For these 526 output values, the overall predictive quality of the model was correct. The model's predictive quality was good as its efficiency value was correct: 0.51. The Root Mean Square Error of Prediction error was quite high, 24%. The bias was positive (5.0%), so the model slightly underestimated final eyespot incidences.

## Discussion

### Interests and Limitations of IPSIM-Wheat-Eyespot

Several studies have been conducted to analyse the effects of cropping practices on the development of eyespot on wheat [10], [29]. However, only one statistical model had been developed in order to predict the incidence of eyespot as a function of few cropping practices [29]. IPSIM-Wheat-Eyespot offers new possibilities for the design of innovative cropping systems since it is the first functional model to encompass simultaneously the effects of soil, climate and the cropping system and to represent the effects of interactions among these many factors.

The development of IPSIM-Wheat-Eyespot was made possible using (1) a schematic representation of the relationships between cropping practices, the production situation and injuries, (2) the translation of this conceptual scheme into a simulation model, and (3) a combination of data from a wide range of production situations (many regions and years) to test its predictive quality.

#### 1. Conceptual Bases of IPSIM-Wheat-Eyespot

The conceptual scheme of IPSIM-Wheat-Eyespot is innovative because i) it encompasses a temporal scale longer than the cropping season (effect of the crop sequence in interaction with tillage over two years); ii) the main cropping practices that can affect the disease are represented; iii) interactions between practices as well as interactions between practices and climate are taken into account. As compared to the conceptual scheme of IPSIM [33], the spatial scale considered was limited to the field because of the lack of interactions at larger scales. In addition, the conceptual scheme of IPSIM-Wheat-Eyespot does not take into account socio-economic drivers, farmer's goals and cognition since it does not aim at simulating decisions. However, this original conceptual model can help design innovative cropping systems less susceptible to eyespot. The information provided by IPSIM-Wheat-Eyespot should be combined with other sources of information (references, other models, or expertise) in order to design new cropping systems, especially since damage (*i.e.* crop loss) caused by the disease are not represented.

#### 2. Hierarchical Tree of Attributes and Aggregating Tables

The qualitative nature of the DEX method is well suited to the modelling of complex systems for which no high level of precision is required. The DEXi software tool [36] offered a suitable environment for the organisation of available knowledge and a rapid development of IPSIM-Wheat-Eyespot. The main breakthrough of the IPSIM platform is to allow the handling of complexity in a simple way [33]. The work presented in this paper provides a proof of concept for this innovative modelling approach in the field of crop protection for a single disease. A major innovation of this modelling approach is to be able to aggregate attributes of different natures (e.g. cultivar choice, a nominal variable and fertilisation rate, a quantitative variable) to describe the impact of various components of cropping systems and their interactions on eyespot incidence. IPSIM-Wheat-Eyespot is actually the first model which can overcome the lack of data on the relationships between cropping practices and a single pest in a given production situation to help design strategies to control the disease. The qualitative DEXi approach may lead to a loss of precision and sensitivity in the developed model [67]. Increasing the number of attribute scales at the top of the decision tree could be a way to improve the sensitivity [68]. However, it results in more complicated aggregating tables which are consequently more difficult to define. Due to the tremendous complexity of interactions between cropping practices and the production situation, a smaller number of indicator states have been chosen to keep the representation of the complex underlying mechanisms as simple as possible. A correct definition of aggregating tables is of primary importance in DEXi models [68]. The choice of the nature and the number of qualitative scales is also crucial and will partly determine the quality of prediction. The choices of both aggregating tables and qualitative scales of attributes have to be explicit and traceable. Indeed, the scales and the aggregating tables used for the attributes of IPSIM-Wheat-Eyespot could not be determined in a generic way but have been specifically defined according to experimental results, models available in the literature and expert knowledge if need be. Unfortunately, literature to analyse some attributes may not exist, lack certain features, or controversial. For instance, the impact of soil type on eyespot incidence is very poorly described in the international literature and the relationships between tillage and eyespot are subject to much controversy [43]. For these cases, expert knowledge had to be used to complete some aggregating tables. In addition, the model runs using simple “if–then” rules, which are “shallow” in the sense that they only define direct relationships between conditions and consequences, but do not represent any “deeper” (or mechanistic) biological, physical, chemical processes [69]. Since the early stages of development of IPSIM-Wheat-Eyespot, it has been clear that precision was not an objective of the model. It appears more important to focus on accuracy rather than precision when modelling such a complex system.

Table 2 reveals the overall behaviour of IPSIM-Wheat-Eyespot. This is also an additional value of the IPSIM approach: the model is transparent and can be easily discussed. For instance, it is clear that the overall effect of fungicide on the disease is low (21%). This is because fungicide does not always control the disease efficiently [59]. The main factor influencing the disease is the spring weather (38%). This is consistent with Matusinsky et al. [43] who showed that the disease was very dependent on the climatic conditions during spring.

#### 3. Predictive Quality of IPSIM-Wheat-Eyespot

The quality of the analysis of the IPSIM-Wheat-Eyespot predictive quality not only depends on the model itself (hierarchical structure of attributes and aggregating tables) but also on the diversity of the data used, which must reflect a wide range of production situations. These data should represent a variety of soil, climate and cropping practices, but also of final incidences. The dataset used in this study satisfied the three former conditions, but did not fully satisfy the latter. The observed final eyespot incidences were generally quite low, so the predictive quality of IPSIM-Wheat-Eyespot could not be extensively evaluated for high levels of incidence.

The main difference with other models is that IPSIM-Wheat-Eyespot is based on qualitative variables and not quantitative ones. The use of qualitative data requires greater attention to the description of the adopted hypotheses, because qualitative data are more difficult to interpret objectively [68]. This is particularly the case for the transformation of quantitative variables that have to be translated into qualitative input of IPSIM-Wheat-Eyespot (e.g. sowing density expressed in kg/ha or number of seeds m^−2^ and translated into “low”, “normal” or “high”). Thus, IPSIM-Wheat-Eyespot can be used in 2 ways. On the one hand, some users can provide directly qualitative basic attributes (i.e. input variables of the model) if they want to test the performance of some technical options in given production situation. On the other hand, other users might want to run the model for real or putative situations where both the production situation and cropping practices are characterised with quantitative, qualitative or nominative data. In this case, an algorithm should be developed in order to rigorously translate these data into appropriate basic attributes based on national or international official references (e.g. a given cultivar will be classified as “very susceptible to susceptible”; “moderately susceptible”; or “resistant” according to official national or international seed classification); regional references (e.g. a given sowing date will be classified as “early”; “normal”; “late” as a function of regional references established by extension services); knowledge available in the literature (e.g. a given crop will be classified as “host”, “non-host”, or “risk-amplifying crop” according to published scientific articles); references produced by models (e.g. a given weather scenario can be classified as “very favourable”; “favourable”; or “unfavourable” according to a published model).

IPSIM-Wheat-Eyespot proved to fairly represent the variability of the 526 “site-years” used to test its predictive quality. This indicates that the model is already operational and can represent the effects of a wide range of production situations*cropping practices combinations for eyespot epidemics to help design cropping systems less susceptible to the disease. This is remarkable since, unlike most models, no fitting procedure was used.

### Prospects

#### 1. Improvements to the Model

Further refinements could be added in the future. They should keep the balance between: i) modelling of the effects of cropping practices and the production situation on eyespot epidemics as accurately as possible, and ii) keeping the model as simple as possible. In addition to the design of a model, the approach presented in this article allowed us to structure the available knowledge in the literature about the effects of cropping practices and the production situation on eyespot epidemics (Table 1). Aggregating tables derived from Table 1 could be easily adapted according to future advances in the knowledge of underlying mechanisms responsible for the disease. In the same way, the model structure could easily be modified to integrate new knowledge. For instance, the model does not yet take into account the effects of cultivar mixtures, whereas some authors have described a reduction of eyespot by cultivar mixtures [70]–[72]. However, this cropping practices is not currently widespread, data are sparse and there is no consensus in the literature on this matter.

IPSIM-Wheat-Eyespot requires the provision of qualitative basic attributes. This is a benefit for the *ex-ante* design of innovative cropping systems. However, this requires translating nominative or quantitative variables used to describe cropping practices and the production situation into *ad hoc* qualitative variables. In order to avoid subjectivity when translating these variables, some reference values have to be used. Such values were gathered for several French regions (data not shown) in order to design an algorithm that translates nominative or quantitative variables describing cropping practices and the production situation into relevant basic attributes of the model. This algorithm can be easily adapted to any location where wheat is grown and eyespot is present, provided that relevant reference values are available. At last, aggregating tables could be adjusted to improve IPSIM-Wheat-Eyespot's predictive quality using statistical procedures, as done for parameter estimation for quantitative models.

#### 2. Future Use of the Model

The main breakthrough of the IPSIM framework, with a simple hierarchical aggregative structure, is to allow the handling of complexity in a simple way. The input variables of models developed with IPSIM, such as IPSIM-Wheat-Eyespot, are easily obtained [33]. IPSIM-Wheat-Eyespot will help design cropping systems with a lower risk of eyespot on wheat. In order to do so, simulation plans will be defined to assess the performance of cropping practices in a given production situation with regard to the control of the disease. It is obvious that this simulation work will have to be combined with other sources of information such as other models, expert knowledge, diagnoses in commercial fields or experiments to propose innovative sustainable cropping systems.

The model, along with the interface that translates nominative and quantitative variables into relevant qualitative input variables for IPSIM-Wheat-Eyespot (Microsoft® Office Excel 2003), is now available upon request. This model can now be used as a communication, organisation, training and teaching tool for researchers, extension engineers, advisers, teachers or even farmers. Appropriation and adaptation of the model by technicians, advisers or farmers could be useful to exchange knowledge and experience (building up from their technical know-how).

The model presented in this paper only takes into account one pest among the biocenosis of a wheat field. Nevertheless, it is necessary to consider the entirety of the major pests when designing cropping systems because farmers have to manage combinations of pest populations, leading to injury profiles, which can in turn lead to quantitative or qualitative damage and ultimately economic losses. In addition to being a model specific to a given disease, IPSIM-Wheat-Eyespot can also be seen as the first sub-model of IPSIM-Wheat, a model that will predict injury profiles on wheat as a function of cropping practices and the production situation.

## Supporting Information

Figure S1 **Aggregating table used for the calculation of the value of the aggregative attribute “Final incidence of Eyespot” (screenshot of the DEXi software).**
(TIF)

Figure S2 **Aggregating table used for the calculation of the value of the aggregative attribute “Effects of cropping practices” (screenshot of the DEXi software).**
(TIF)

Figure S3 **Aggregating table used for the calculation of the value of the aggregative attribute “Effects of soil and climate” (screenshot of the DEXi software).**
(TIF)

Figure S4 **Aggregating table used for the calculation of the value of the aggregative attribute “Primary inoculum management: interaction between crop sequence and tillage” (screenshot of the DEXi software).**
(TIF)

Figure S5 **Aggregating table used for the calculation of the value of the aggregative attribute “Mitigation through crop status” (screenshot of the DEXi software).**
(TIF)

Figure S6 **Aggregating table used for the calculation of the value of the aggregative attribute “Climate” (screenshot of the DEXi software).**
(TIF)
