# Supplementary figures and images for: Injury Profile SIMulator, a Qualitative Aggregative Modelling Framework to Predict Injury Profile as a Function of Cropping Practices, and Abiotic and Biotic Environment. II. Proof of Concept: Design of IPSIM-Wheat-Eyespot
Source: PLoS One. 2013 Oct 16;8(10):e75829. doi: 10.1371/journal.pone.0075829 (PMC3797717; doi:10.1371/journal.pone.0075829)

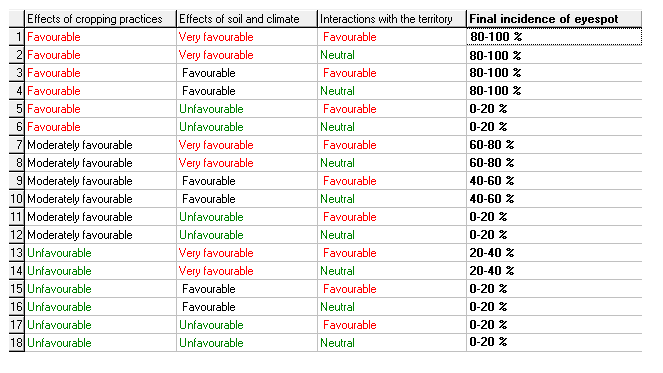

Supplement: Figure S1 — Aggregating table used for the calculation of the value of the aggregative attribute “Final incidence of Eyespot” (screenshot of the DEXi software). (TIF) [file pone.0075829.s001.tif]

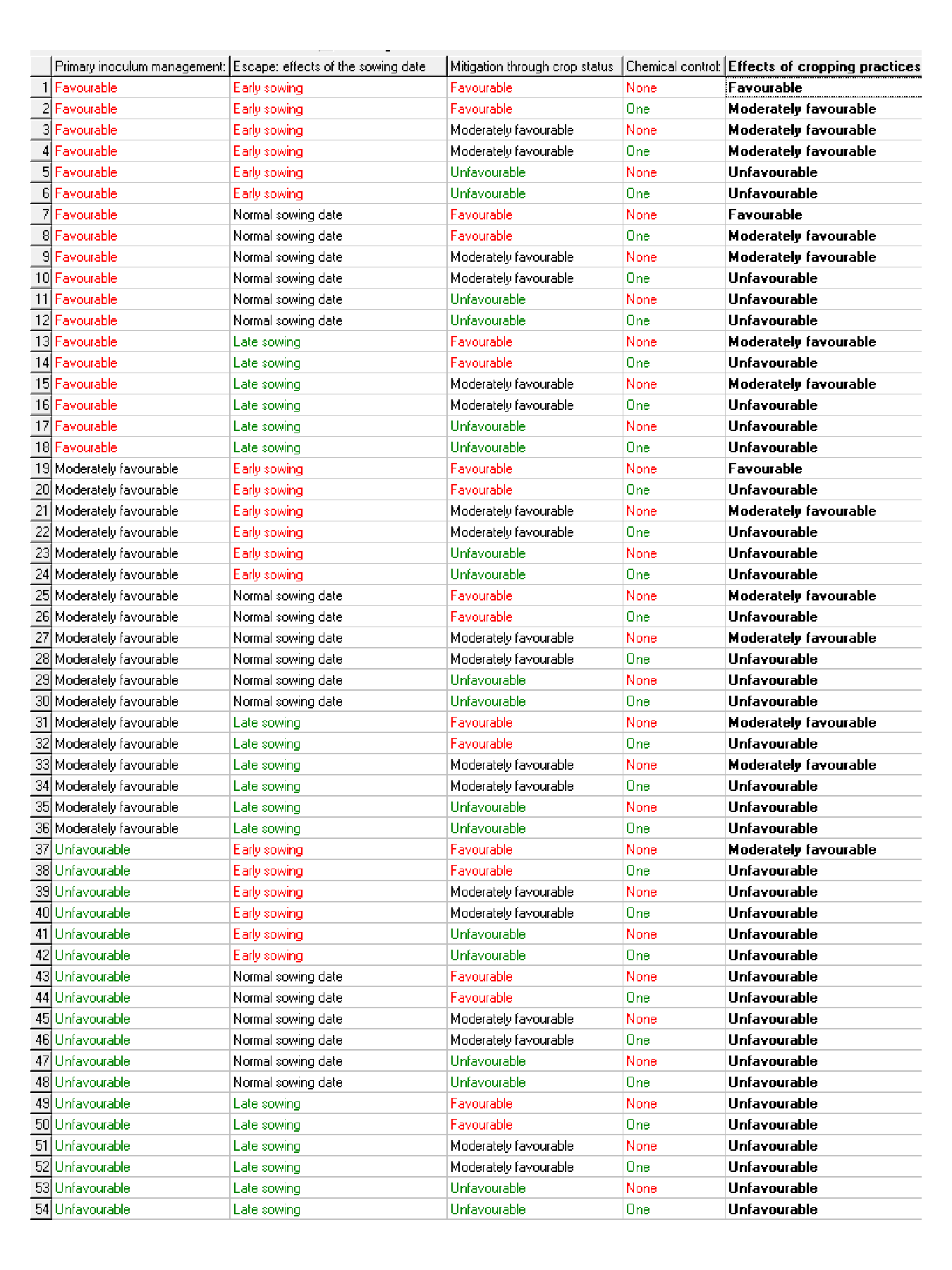

Supplement: Figure S2 — Aggregating table used for the calculation of the value of the aggregative attribute “Effects of cropping practices” (screenshot of the DEXi software). (TIF) [file pone.0075829.s002.tif]

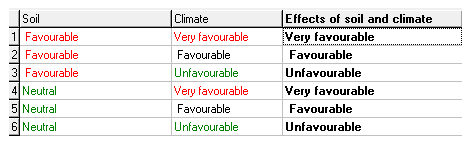

Supplement: Figure S3 — Aggregating table used for the calculation of the value of the aggregative attribute “Effects of soil and climate” (screenshot of the DEXi software). (TIF) [file pone.0075829.s003.tif]

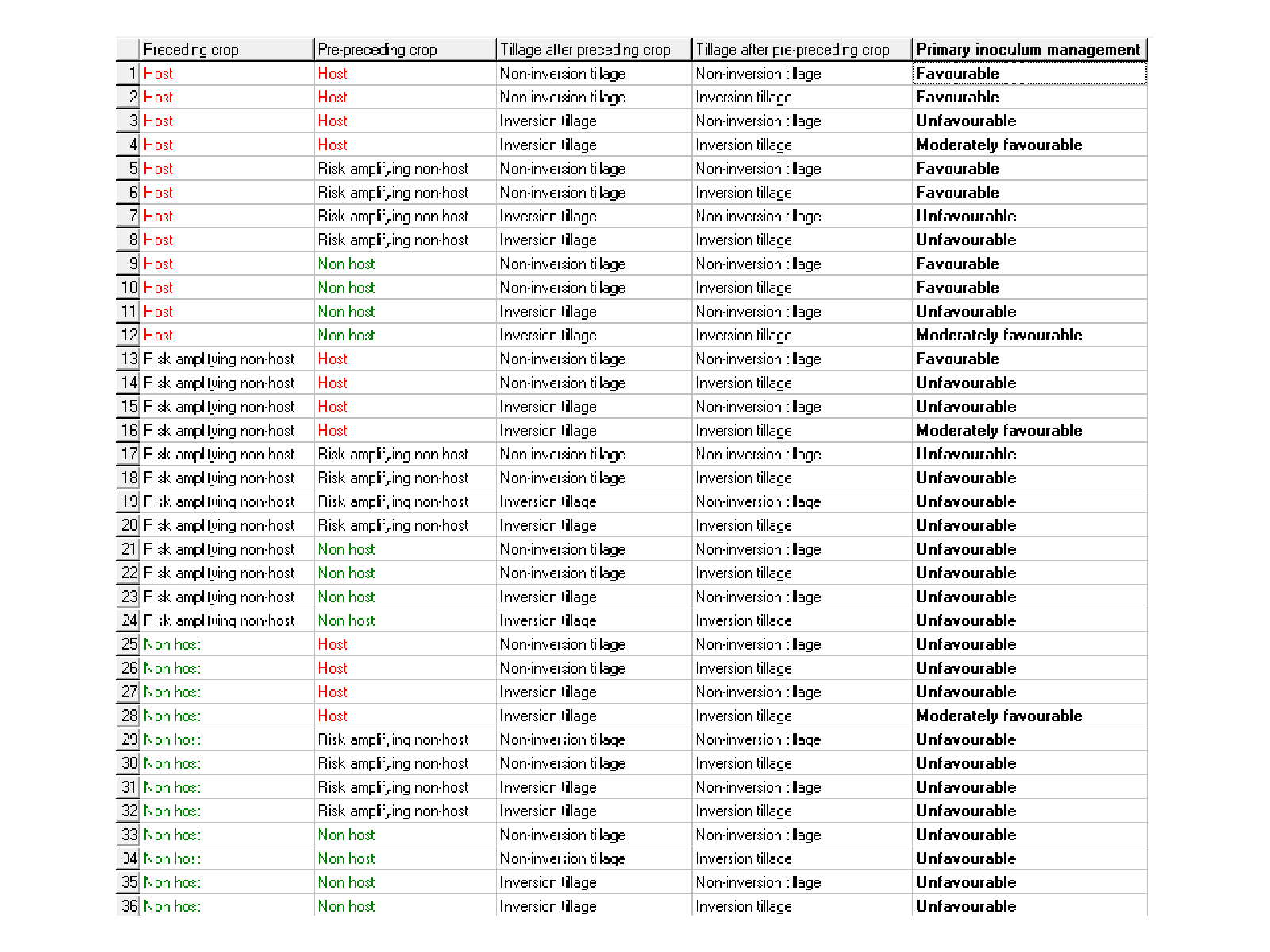

Supplement: Figure S4 — Aggregating table used for the calculation of the value of the aggregative attribute “Primary inoculum management: interaction between crop sequence and tillage” (screenshot of the DEXi software). (TIF) [file pone.0075829.s004.tif]

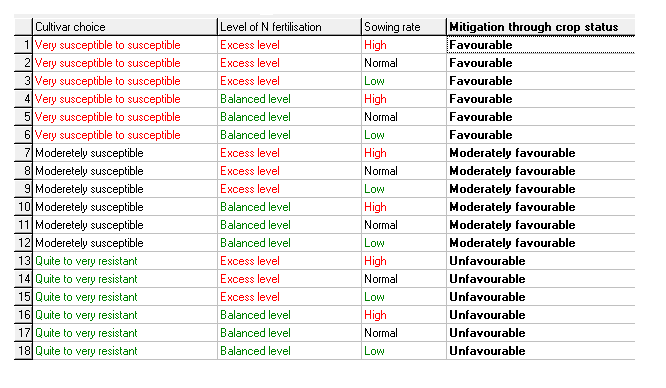

Supplement: Figure S5 — Aggregating table used for the calculation of the value of the aggregative attribute “Mitigation through crop status” (screenshot of the DEXi software). (TIF) [file pone.0075829.s005.tif]

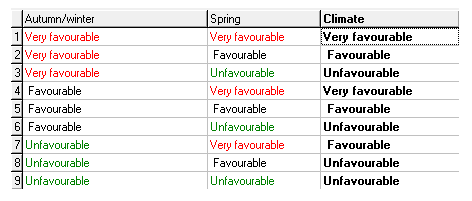

Supplement: Figure S6 — Aggregating table used for the calculation of the value of the aggregative attribute “Climate” (screenshot of the DEXi software). (TIF) [file pone.0075829.s006.tif]
